# Understanding the cost of pharmacy-delivered HIV pre- and post-exposure prophylaxis service delivery in Kenya: findings from pilot studies

**DOI:** 10.1186/s12913-025-13681-x

**Published:** 2025-11-26

**Authors:** Obinna I. Ekwunife, Alexandra P. Kuo, Preetika Banerjee, Shengruo Zhang, Catherine Kiptinness, Victor Omollo, Yilin Chen, Stephanie D. Roche, Josephine Odoyo, Elizabeth Bukusi, Kenneth Ngure, Monisha Sharma, Katrina F. Ortblad

**Affiliations:** 1 Public Health Sciences Division, Fred Hutchinson Cancer Center, 1100 Fairview Ave N, Seattle, WA 98109 USA; 2 Division of Population Health, Department of Medicine, Jacobs School of Medicine and Biomedical Sciences, University at Buffalo, Buffalo, NY USA; 3 Department of Epidemiology, University of Washington, Seattle, USA; 4 Center for Clinical Research, Kenya Medical Research Institute, Thika, Kenya; 5 Kenya Medical Research Institute, Kisumu, Kenya; 6 The Comparative Health Outcomes, Policy and Economics (CHOICE) Institute, School of Pharmacy, University of Washington, Seattle, USA; 7 Department of Global Health, University of Washington, Seattle, USA; 8 Department of Obstetrics and Gynecology, University of Washington, Seattle, USA; 9 Centre for Microbiology Research, Kenya Medical Research Institute, Nairobi, Kenya; 10 School of Public Health, Jomo Kenyatta University of Agriculture and Technology, Nairobi, Kenya

**Keywords:** Microcosting, Differentiated service delivery, HIV prevention, PrEP, Kenya, Pharmacies, Costs, Africa

## Abstract

**Background:**

Private pharmacies are a primary access point for health services in many African countries. Leveraging private-sector pharmacies to deliver HIV prevention services, including pre- and post-exposure prophylaxis (PrEP/PEP), may expand the reach of these services. Understanding delivery costs is necessary to inform scale-up.

**Methods:**

We used data from two pilot studies conducted in Kisumu County, Kenya. In the first pilot, pharmacy providers at 12 pharmacies delivered free PrEP/PEP to eligible clients (≥ 18 years) using a prescribing checklist with remote clinician oversight; PrEP/PEP drugs were donated from government stock. Using microcosting, we estimated the economic and financial costs from the provider’s perspective for: (1) subsidized delivery (donated commodities excluded), and (2) non-subsidized delivery (donated commodities included). We also assessed client willingness to pay for PrEP services at pharmacies using PrEP client survey data. In the second pilot, pharmacy providers at 20 pharmacies delivered HIV testing services. We assessed providers’ anticipated willingness to deliver PrEP services using provider survey data.

**Result:**

From February to July 2022, pharmacies in the first pilot recorded 1,564 PrEP/PEP visits, and initiated 691 clients on PrEP. Among clients eligible to continue PrEP at the pharmacy, 69% (479/691) refilled at least once. We collected 694 surveys from PrEP clients. From March to June 2022, 40 providers in the second pilot completed surveys. The estimated economic (financial) costs per client-month on PrEP were $3.66 ($2.17) USD for subsidized and $13.23 ($11.74) USD for non-subsidized delivery, and for PEP were $3.66 ($2.15) USD for subsidized and $10.75 ($9.24) USD for non-subsidized delivery. Most PrEP clients (83%, 575/691) expressed willingness to pay for pharmacy-delivered PrEP services; the median amount they were willing to pay per visit was $3.30 USD (IQR $1.60-$4.10 USD), which exceeded the median maximum amount providers said they would charge per visit ($2.40 USD, IQR $1.60-$4.10 USD).

**Conclusions:**

When subsidized with drugs from government stock, pharmacies are a low-cost platform for delivering PrEP and PEP services in Kenya. Client out-of-pocket payments could help sustain pharmacy PrEP/PEP delivery at scale, enabling broader coverage of HIV prevention services.

**Supplementary Information:**

The online version contains supplementary material available at 10.1186/s12913-025-13681-x.

## Introduction

In Kenya and many other African countries, HIV incidence continues to exceed both national HIV prevention targets and levels needed for epidemic control [1]. For example, whereas Kenya’s current goal is to have under 11,000 new HIV infections annually, it reported 17,000 new infections in 2023 [1, 2]. Differentiated delivery of HIV prevention services, including in non-clinical settings, can enhance reach and accessibility [3, 4]. Private pharmacies are one promising delivery platform, as they have several advantages over public clinics, including greater privacy, less HIV-associated stigma, and expedited service delivery [5]. Delivering pre- and post-exposure prophylaxis (PrEP and PEP) at private pharmacies has the potential to reach new populations and achieve the last mile of HIV epidemic control in Kenya [6].

In Kenya, private pharmacies are typically community-based and operated by individuals or private companies, whereas public pharmacies are government-run, located within public clinics, and generally offer lower-cost medications. Private pharmacies already play a vital role in the delivery of sexual and reproductive health services, particularly for young women. Their convenience and confidentiality make them a preferred source for contraception, especially emergency contraception. Approximately 94% of women who purchase emergency contraception do so from private pharmacies [7, 8], and they are frequently the first point of contact for individuals seeking sexual and reproductive health products [9, 10].

Kenya has been a leader in developing and evaluating pharmacy-based PrEP and PEP delivery models that utilize existing staff—pharmacists and pharmaceutical technologists—or introduce new staff to reach key populations (e.g., nurse navigators). In these models, trained providers deliver PrEP and PEP services to eligible clients using a prescribing checklist with oversight by a remote prescribing clinician [11]; clients who do not meet checklist criteria (e.g., those reporting a history of kidney issues) are referred to nearby public clinics for further eligibility evaluation. To date, three pilot studies evaluating variations of this model have demonstrated that private pharmacies reach populations who could benefit from PrEP/PEP—including those not typically engaged in clinic-based services (e.g., young men, unmarried people)—and achieve levels of PrEP initiation and continuation comparable to those observed at public clinics [12–14]. Additionally, qualitative studies of current and prospective pharmacy PrEP clients suggest that pharmacy-based PrEP delivery is highly acceptable to clients and, for some, preferable to public clinic-based delivery [4, 15].

To inform the implementation, scale-up, and sustainability of pharmacy-delivered PrEP and PEP services, we need to understand the incremental costs associated with this delivery approach, including compensation amounts that might motivate pharmacy providers to deliver these services and service fees that pharmacy clients might be willing to pay to obtain these services in this setting. Drawing on data from two pilot studies of pharmacy-delivered HIV services in Kenya [13, 16], we sought to estimate the cost of this delivery approach from different payer scenarios and assess willingness to pay for and provide these services among pharmacy clients and providers.

## Methods

### Study setting

This research took place in Kisumu and Kiambu Counties, where population-level HIV prevalence is 19% and 3%, respectively [17]. In each of these regions, there are 171 licensed private pharmacies [18].

### Pilot studies

We used data from two pilot studies of pharmacy-delivered HIV services. To estimate the cost of delivering PrEP and PEP services at pharmacies and client willingness to pay for these services, we used data from the Pharm PrEP Pilot Extension Study (hereafter, “Pilot Extension”). From January to July 2022, this study evaluated a model of pharmacy-delivered PrEP and PEP services at 12 private pharmacies, evenly split between Kiambu and Kisumu Counties. Study details, including primary results, have been published [13, 14]. In brief, pharmacists and pharmaceutical technologists determined PrEP/PEP eligibility using a standardized prescribing checklist and provider-assisted blood-based HIV self-testing (HIVST; Mylan HIV Self-Test kits, Atomo Diagnostics, Australia). Kenya Ministry of Health (MoH) donated the PrEP and PEP drugs from government stock to study pharmacies; however, the study independently procured HIVST kits after MoH stock was depleted. Eligible pharmacy clients (≥ 18 years old) received PrEP/PEP services for free. During the pilot implementation, study pharmacies were compensated—10,000 KES ($86 USD) per month in Kiambu County and 15,000 KES ($129 USD) per month in Kisumu Country—for time spent delivering PrEP/PEP services and use of their space and utilities by a research assistant. This amount was determined following engagement with stakeholders—including pharmacy owners and county health management officers—in each region.

To assess pharmacy provider willingness to deliver PrEP services, we analyzed data from the HIVST Performance Study, which was conducted in Kisumu County from March to June 2022 and whose primary results have been published [16]. In this study, trained pharmacy providers at 20 private pharmacies delivered HIVST services and referred clients to clinic-based PrEP or ART services, as appropriate.

### Data sources

We used diverse data sources—including surveys, study budgets, and government records—to estimate the unit cost of pharmacy-delivered PrEP and PEP services per client month. We surveyed Pilot Extension providers to obtain capital, overhead, and personnel costs, as well as estimates of time spent delivering the following core components of the delivery model: counseling, HIV testing, and drug dispensing. We obtained costs associated with demand generation and provider training from study budgets. We obtained the cost of PrEP and PEP drugs from the government’s medical logistics provider (Kenya Medical Supplies Authority) and of HIVST kits from the study’s implementing partner (Jhpiego). The questionnaire used for collecting cost data is shown in Additional file 1.

To assess willingness to pay for and deliver pharmacy-based PrEP services, we used survey data from Pilot Extension clients who initiated PrEP and from HIVST Performance Study providers who delivered HIV testing services. Willingness-to-pay insights are important for aligning costs with accessibility and informing sustainable pricing or subsidy strategies, as client demand and affordability influence pricing decisions. The questions used for collecting these data are shown in Additional file 1. In the Pilot Extension, clients were asked how much they would be willing to pay to receive HIV testing, counseling, and a 3-month supply of oral PrEP at a pharmacy. In the HIVST Performance Study, providers were asked the minimum and maximum amount they would charge to deliver oral PrEP, PEP, and a bimonthly long-acting PrEP injection if they received all commodities—including the drugs and HIV testing kits—for free and were only responsible for delivering relevant HIV testing, counseling, and supply of PrEP.

### Analysis

We utilized a microcosting (i.e., bottom-up) approach to estimate the average cost of delivering PrEP or PEP per client per month, as outlined in Additional file 2. To derive the cost of training, we included the resources it took to train pharmacy providers on the delivery of PrEP and PEP. For capital items (e.g., computers, printers, cabinets), we used 2022 market prices annualized over a useful life of five years [19, 20]. To calculate the time costs providers spent delivering PrEP and PEP, we used staff annual gross salaries (including fringe benefits) adjusted for the average time providers reported spending on service delivery. To estimate overhead costs (e.g., maintenance, utilities, medical envelopes, gloves, hand sanitizers, administrative personnel), we used the share of provider time dedicated to PrEP and PEP delivery compared to the delivery of other pharmacy products, as reported by providers. We then summed costs across these categories to generate the total costs incurred delivering PrEP or PEP over the pilot duration, and divided total cost by the number of clients-months of PrEP and PEP use [13].

We estimated the average cost of PrEP or PEP delivery per client month for two scenarios: (1) a *subsidized scenario*, which excludes the cost of PrEP, PEP, and HIVST kits presuming these are donated to pharmacies by the government or international funders, and (2) a *non-subsidized scenario*, which includes the cost of these commodities, thus representing the usual set-up for private pharmacies in Kenya, whereby pharmacies procure commodities from suppliers and clients pay for services out-of-pocket. Table 1 provides additional details on the costs included in the different scenarios. Additionally, for each scenario, we estimated the economic cost of delivering PrEP/PEP at pharmacies, and the financial cost, which excludes capital costs (e.g., facility rent, computers, printers, furniture, and annual professional license renewals) [21, 22]. All analyses were conducted from the pharmacy provider’s perspective using 2022 United States Dollars (USD).

**Table 1:** Items included in the various scenarios for the cost analysis of pharmacy-delivered PrEP/PEP services

	Subsidized PrEP/PEP delivery		Non-subsidized PrEP/PEP delivery	
	Economic cost	Financial cost	Economic cost	Financial cost
Capital costs (e.g., computers, printers, rent)	✓		✓	
Provider training costs	✓	✓	✓	✓
Demand creation activities	✓	✓	✓	✓
Personnel costs	✓	✓	✓	✓
Overhead costs (e.g., maintenance and repairs)	✓	✓	✓	✓
Supplies				
*PrEP or PEP medication*			✓	✓
*HIV rapid diagnostic test kits*			✓	✓
*Administrative supplies*	✓	✓	✓	✓
*Medical waste*	✓	✓	✓	✓

We report willingness to pay/deliver and PrEP initiation and continuation outcomes using summary statistics.

### Sensitivity analyses

To assess how changes in provider time spent delivering PrEP/PEP and PrEP client volume influenced our unit cost estimates, we conducted one-way sensitivity analyses. First, recognizing the possibility that providers may have under- or over-reported the amount of time they spent delivering PrEP/PEP services, we explored scenarios that varied provider time by +/- 20%. Since PrEP client volume could vary by geographic region and/or season, we also explored scenarios that varied client volume by +/- 20%.

Additionally, pharmacy providers reported long working hours, with a median of 13 h per day (interquartile range [IQR] 12–14), based on a six-day workweek [16]. Pharmacy technologists faced low remuneration, earning a median annual salary of 420,000 KES (IQR 240,000–538,000 KES) or $3,612 USD (IQR $2,064–$4,263 USD), with most lacking benefits such as vacation and sick leave. To assess the impact of improved working conditions on cost estimates, we used primary data from other pharmacies in Kenya. In pharmacies included in a cluster randomized controlled trial, providers reported a five-day workweek, nine-hour workdays, 40 days of combined vacation and sick leave annually, and an average annual remuneration of 540,000 KES ($4,644 USD) for pharmacy technologists.

## Results

From February to July 2022, pharmacies in the Pilot Extension initiated 691 clients on PrEP and 162 clients on PEP. A total of 479 PrEP clients returned for follow-up after one month, and 197 returned after three months. Additionally, 35 PEP clients returned for repeat PEP services after completing their 28-day course, resulting in a total of 1,564 PrEP/PEP visits.

Among clients who initiated pharmacy PrEP or PEP, the median age was 25 years (interquartile range [IQR] 22–31) for PrEP clients and 25 years (IQR 22–29) for PEP clients, and less than a quarter of these clients were married (PrEP clients: 23%, 160/691; PEP clients: 17%, 28/162), Table 2.

From March to June 2022, 40 pharmacy providers in the HIVST Performance Study completed surveys. The median provider age was 31 years (IQR 27–37), 40% (16/40) were women, and 42% (17/40) were the pharmacy owner, Table 3. Additionally, the median years of experience in the pharmacy profession was 6 (IQR 4–10).

**Table 2:** Characteristics of pharmacy clients who initiated PrEP or PEP in the pharm PrEP pilot extension study

**Characteristic**	**Pilot Extension Study** ^**1**^	
	**PrEP Clients** **(** ***n*** **= 691)** ^**2**^	**PEP Clients** **(** ***n*** **= 162)**
Age, median (IQR)	25 (22–31)	25 (22–29)
Female	382 (55%)	68 (42%)
< 25 years	331 (48%)	79 (49%)
Education: University degree	155 (22%)	69 (43%)
Married	160 (23%)	28 (17%)
Monthly household income, median US$ (IQR)	86 (43–172)	129 (6–258)
**Characteristic**	**HIVST Performance Study** ^**3**^ **Pharmacy providers** **(** ***n*** **= 40)**	
Age, median (IQR)	31 (27–37)	
Female	16 (40%)	
Pharmacy owner	17 (42%)	
Years in pharmacy profession, median (IQR)	6 (4–10)	
Days working at pharmacy per week, median (IQR)	6 (6–7)	

^1^Roche SD, Omollo V, Mogere P, Asewe M, Gakuo S, Banerjee P, et al. Pharmacy-based PrEP delivery in Kenya: findings from a pilot study extension. In Seattle, Washington; 2023. Available from: https://www.croiconference.org/abstract/pharmacy-based-prep-delivery-in-kenya-findings-from-a-pilot-study-extension/. ^2^This number includes 30 PEP clients who subsequently transitioned to PrEP

**Table 3:** Characteristics of pharmacy providers who delivered HIV testing services in the HIVST performance study

Characteristic	HIVST Performance Study^3^ Pharmacy providers (*n* = 40)
Age, median (IQR)	31 (27–37)
Female	16 (40%)
Pharmacy owner	17 (42%)
Years in pharmacy profession, median (IQR)	6 (4–10)
Days working at pharmacy per week, median (IQR)	6 (6–7)

^3^Ortblad KF, Kwach B, Zhang S, Asewe M, Ongwen PA, Malen RC, et al. Measuring the performance of HIV self-testing at private pharmacies in Kenya: a cross‐sectional study. J Int AIDS Soc. 2023 Oct;26 [10]:e26177. 1 KES = $0.0086

### Time spent delivering core components of PrEP/PEP services

To deliver all core components of PrEP/PEP services, pharmacy providers reported taking an average of 56 min (standard deviation [SD] 31) for a PrEP initiation visit, 35 min (SD 12) for a PrEP follow-up visit, and 45 min (SD 17) for a PEP visit (including initiation and follow-up), Table 4. Conducting HIV testing was the most time-consuming activity, representing, on average, ~ 40% of total delivery time for each visit type. The next most time-consuming components were HIV risk screening and drug dispensing, each accounting for ~ 15% to ~ 20% of the total delivery time, on average.

**Table 4:** Average provider time spent per client visit on each core component of PrEP and PEP delivery

Core component of delivery model	Minutes Average (standard deviation)		
	PrEP initiation visit	PrEP follow-up visit	PEP visit
Screening for HIV risk	12 (6)	5 (2)	7 (4)
Counselling on PrEP/PEP	6 (3)	7 (4)	4 (2)
HIV testing^1^	21 (9)	18 (6)	19 (4)
Drug dispensing	10 (9)	7 (7)	9 (12)
Additional counselling^2^	-	-	8 (8)
Documentation	9 (13)	6 (6)	4 (2)
**Total visit duration**	**56 (31)**	**35 (12)**	**45 (17)**

^1^Includes assisting clients with HIV self-test administration and confirming the test result

^2^At follow-up visits, PEP clients who tested HIV-negative were counseled on the option to transition to PrEP

### Cost of pharmacy-delivered oral PrEP and PEP services in subsidized scenario

Table 5 summarizes the estimated economic and financial costs for pharmacy-delivered PrEP and PEP services under the two scenarios of interest. In the subsidized delivery scenario, estimated economic costs were $3.66 USD per client month for PrEP and $3.66 USD per person-month for PEP; the primary cost drivers were capital (41% for both PrEP and PEP) and personnel (39% for both PrEP and PEP). Estimated financial costs for the subsidized delivery scenario were $2.17 USD per person-month for PrEP and $2.15 USD per person-month for PEP; the primary cost driver was personnel (66% for PrEP and 67% for PEP).

### Cost of pharmacy-delivered oral PrEP and PEP services in non-subsidized scenario

In the non-subsidized delivery scenario, the estimated economic costs were $13.23 USD per person-month for PrEP and $10.75 USD per person-month for PEP. The primary cost drivers were commodities—specifically drugs and HIVST kits—which accounted for 45% and 27% of PrEP costs, and 32% and 34% of PEP costs, respectively. Capital costs also contributed significantly, comprising 11% of PrEP costs and 14% of PEP costs. Estimated financial costs for the non-subsidized delivery scenario were $11.74 USD per person-month for PrEP and $9.24 USD per person-month for PEP; the primary cost drivers were drugs (51% for PrEP and 37% for PEP) and HIVST kits (31% for PrEP and 39% for PEP).

**Table 5:** The estimated economic and financial costs of pharmacy-delivered PrEP and PEP services

	Subsidized delivery						Non-subsidized delivery					
	PrEP services			PEP services			PrEP services			PEP services		
	Total cost	Unit cost (*n* = 1,367)	% of total	Total cost	Unit cost (*n* = 197)	% of total	Total cost	Unit cost (*n* = 1,367)	% of total	Total cost	Unit cost (*n* = 197)	% of total
**Economic costs (2022 USD)**												
Capital	$2040.70	$1.49	41%	$297.06	$1.51	41%	$2040.70	$1.49	11%	$297.06	$1.51	14%
Training	$76.91	$0.06	2%	$11.08	$0.06	2%	$76.91	$0.06	0%	$11.08	$0.06	1%
Demand creation	$521.58	$038	10%	$65.70	$0.33	9%	$521.58	$0.38	3%	$65.70	$0.33	3%
Personnel	$1943.76	$1.42	39%	$282.36	$1.43	39%	$1943.76	$1.42	11%	$282.36	$1.43	13%
Overhead	$222.08	$0.16	4%	$32.19	$0.16	4%	$222.08	$0.16	1%	$32.19	$0.16	2%
Supplies												
*PrEP/PEP*	$0.00	$0.00	0%	$0.00	$0.00	0%	$8119.98	$5.94	45%	$680.83	$3.46	32%
*HIVST kits*	$0.00	$0.00	0%	$0.00	$0.00	0%	$4966.31	$3.63	27%	$715.70	$3.63	34%
*Admin supplies*	$181.98	$0.13	4%	$30.00	$0.15	4%	$181.98	$0.13	1%	$30.00	$0.15	1%
*Medical waste*	$14.43	$0.01	0%	$2.08	$0.01	0%	$14.43	$0.01	0%	$2.08	$0.01	0%
**Total**	**$5**,**001.45**	**$3.66**	**100%**	**$720.48**	**$3.66**	**100%**	**$18**,**087.74**	**$13.23**	**100%**	**$2**,**117.02**	**$10.75**	**100%**
**Financial costs (2022 USD)**												
Capital	$0.00	$0.00	0%	$0.00	$0.00	0%	$0.00	$0.00	0%	$0.00	$0.00	0%
Training	$76.91	$0.06	3%	$11.08	$0.06	3%	$76.91	$0.06	0%	$11.08	$0.06	1%
Demand creation	$521.58	$0.38	18%	$65.70	$0.33	16%	$521.58	$0.38	3%	$65.70	$0.33	4%
Personnel	$1943.76	$1.42	66%	$282.36	$1.43	67%	$1943.76	$1.42	12%	$282.36	$1.43	16%
Overhead	$222.08	$0.16	7%	$32.19	$0.16	8%	$222.08	$0.16	1%	$32.19	$0.16	2%
Supplies												
*PrEP/PEP*	$0.00	$0.00	0%	$0.00	$0.00	0%	$8119.98	$5.94	51%	$680.83	$3.46	37%
*HIVST kits*	$0.00	$0.00	0%	$0.00	$0.00	0%	$4966.31	$3.63	31%	$715.70	$3.63	39%
*Admin supplies*	$181.98	$0.13	6%	$30.00	$0.15	7%	$181.98	$0.13	1%	$30.00	$0.15	2%
*Medical waste*	$14.43	$0.01	0%	$2.08	$0.01	0%	$14.43	$0.01	0%	$2.08	$0.01	0%
**Total**	**$2**,**960.75**	**$2.17**	**100%**	**$423.42**	**$2.15**	**100%**	**$16**,**047.74**	**$11.74**	**100%**	**$1**,**819.95**	**$9.24**	**100%**

**Abbreviations**: Admin – Administrative; PrEP – HIV pre-exposure prophylaxis; PEP – HIV post-exposure prophylaxis

### Sensitivity analyses

Sensitivity analyses indicated that, for both PrEP and PEP, the economic and financial costs per person-month were only marginally influenced by uncertainties in provider time and client volume. However, if we consider using primary data from other pharmacies in Kenya with improved labor practices, the estimated economic (financial) costs per client-month on PrEP increases by 20% (30%) for subsidized and 7% (7%) for non-subsidized delivery, and for PEP increases by 20% (30%) for subsidized and 8% (9%) for non-subsidized delivery. Additional file 1.

### Willingness to pay for or provide pharmacy-delivered PrEP services

**Fig. 1:**
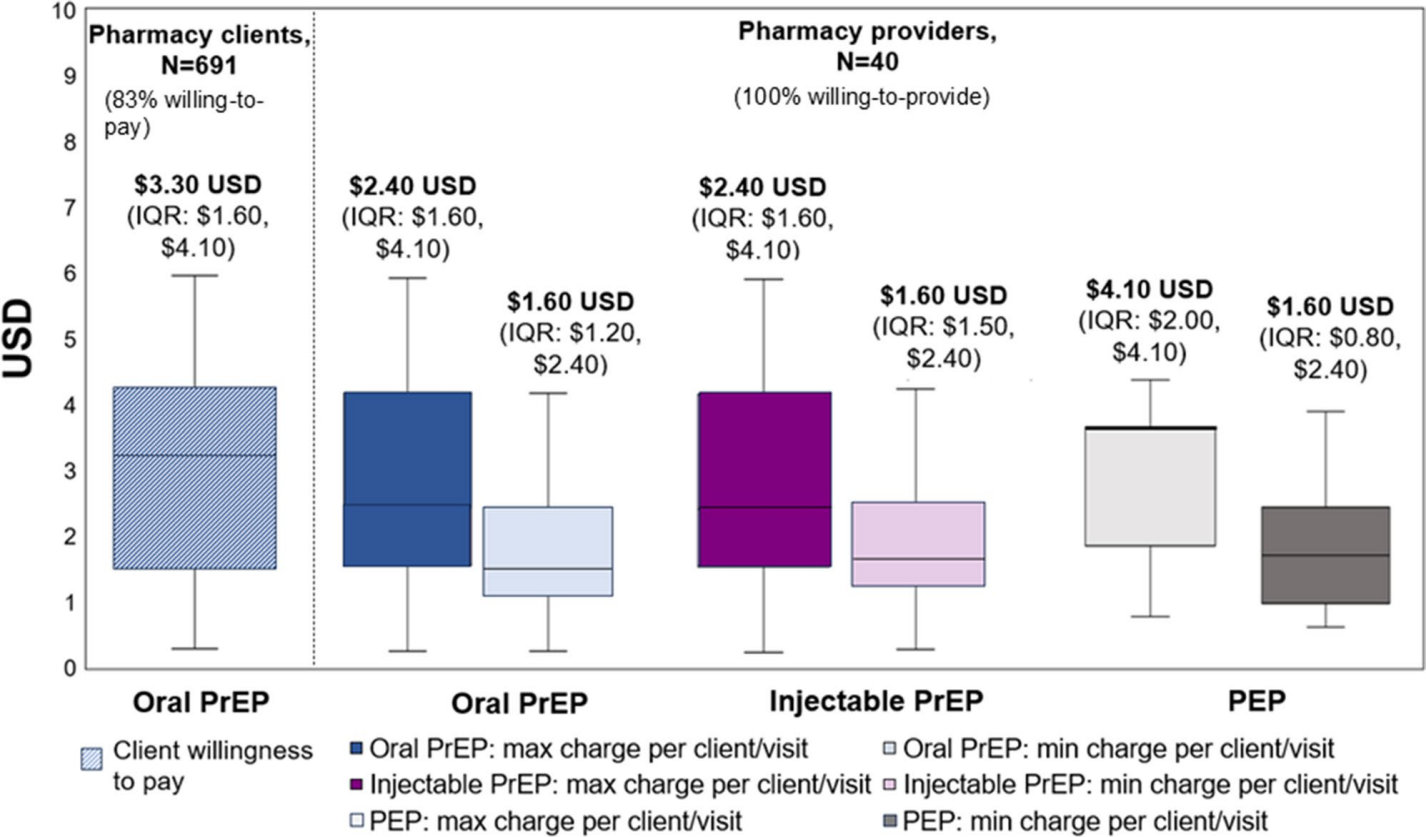
Median amount PrEP clients were willing to pay, and pharmacy providers were willing to charge, for different HIV prevention products at pharmacies in US dollars

Most Pilot Extension clients who received free oral PrEP services in the Pilot Extension (83%, 575/691) said they would be willing to pay to get PrEP at a pharmacy in the future. Additionally, all pharmacy providers (40/40) in the HIVST Performance Study expressed willingness to deliver oral PrEP, injectable PrEP, and PEP services in the future. The median amount PrEP clients said they were willing to pay for pharmacy-based PrEP services—$3.30 USD (IQR $1.60 to $4.10 USD) —exceeded the median maximum amount providers said they would charge for this service ($2.40 USD, IQR $1.60-$4.10), Fig. 1. Across the different HIV prevention products, we asked about—oral PrEP, injectable PrEP, and PEP—there was little variation in what providers said they would be willing to charge if they were provided with the drugs and HIV testing kits for free.

## Discussion

To our knowledge, this study is the first to estimate the cost of delivering HIV PrEP and PEP services in private pharmacies in sub-Saharan Africa. Using data from two pilot studies of pharmacy-based PrEP and PEP delivery in Kenya, we found that pharmacy-delivered PrEP and PEP services provide a low-cost model for expanding HIV prevention efforts compared to cost of delivery in clinic setting. Additionally, most pharmacy clients were willing to pay more than the estimated financial cost of service provision in a subsidized delivery scenario—where commodities were provided to pharmacies at no charge—suggesting optimism for a financial case to make PrEP and PEP available at pharmacies. Further, in a subsidized delivery scenario, pharmacy providers were willing to offer PrEP and PEP at a lower price than what clients were willing to pay, which reinforces the feasibility of the approach in Kenya and other similar countries.

Compared to several clinic-based PrEP delivery programs in Kenya, the economic cost of delivering PrEP and PEP at private pharmacies in the Pilot Extension study was lower. Specifically, the economic cost of non-subsidized PrEP delivery in private pharmacies was $13.23 USD per person-month, whereas this same cost was $21.32 USD in public HIV clinics [23], $26.52 USD at maternal and child health clinics [24], and $28.92 USD in family planning clinic [25]. When we compare the cost drivers of these different delivery settings, we find that personnel costs are substantially lower in the pharmacy model ($1.42 USD per person-month) compared to the clinic model, where personnel costs range from ~$9 USD to $11 USD per person-month [23, 24]. These differences in personnel costs at the pharmacy versus the clinic are largely attributable to differences in the number of providers serving clients—typically only one provider serves clients at the pharmacy while many serve clients at the clinic—and differences in provider annual salaries—with pharmacy providers often having lower salaries than more highly-trained clinic-based clinicians and nurses—in these delivery settings. Other factors that contribute to differences in these cost estimates include additional PrEP implementation activities at the clinics that are not ongoing at the pharmacies—such as clinic-wide health talks and support groups for serodiscordant couples—as well as differing overhead costs, which can be >20 times higher at the clinics compared to the pharmacies.

Clients in our pilot study were willing to pay for pharmacy-delivered oral PrEP—despite not being charged for the service during the study—and were willing to pay more than pharmacy providers indicated they would charge, assuming they received the supporting commodities for free from the government. Given that Kenyan citizens have an average monthly income of ~ 20,123 KES (~$133 USD per month or ~$4.40 USD per day), these willingness-to-pay results suggest that pharmacy clients—especially those in middle and upper socioeconomic classes—could likely afford pharmacy-based PrEP [26] and that out-of-pocket client PrEP payments could potentially offset provider PrEP costs and sustain the approach. Additionally, client payments for PrEP may offer other benefits, such as increased service utilization—since clients are more likely to engage in services they have financially invested in [27]—and improved service quality, from providers seeking repeat business. The long-term sustainability of this model, however, likely depends on the continued free supply of PrEP commodities to private-sector pharmacies. Recent shifts in the global HIV funding landscape, including PEPFAR’s narrowed focus on prevention services for pregnant and lactating individuals, are likely to have far-reaching implications for PrEP and PEP commodity availability [28]. This change places additional financial strain on MoHs, who must now make difficult decisions regarding resource allocation. As prevention commodities may not rank as a top priority amid competing treatment demands, access to subsidized PrEP and PEP could be significantly constrained, particularly in settings where domestic financing is limited. While this may present challenges in a shifting HIV funding landscape [29], it also presents an opportunity to potentially offset personnel costs—costs that could be covered by clients in this novel delivery approach.

Although the amounts clients were willing to pay for PrEP and providers were willing to charge do not reflect real-life pricing, the willingness-to-pay (WTP) and willingness-to-charge estimates—based on the assumption of a public-private partnership with pharmacies—offer valuable insights into perceived affordability and acceptable service charges. These self-reported figures help gauge demand-side expectations and provider considerations, which are important for understanding potential barriers to service uptake in a pharmacy setting. While government budget allocations and MoH pricing targets are essential for long-term program planning, they may not fully capture client perceptions of value or the economic realities of private-sector service provision. Also, in the case of long-acting injectable agents such as lenacapavir being introduced in Kenya, which require administration only once every six months, it may be valuable to assess whether pharmacy providers’ willingness to offer such services would be higher than currently reported, given the limited frequency of client visits. Our findings contribute to broader policy discussions by offering a bottom-up perspective that can inform subsidy strategies, cost-sharing models, and pricing frameworks to support program sustainability.

A threat to the scalability of pharmacy-delivered PrEP and PEP is the time burden on both clients and providers—each of whom values quick services at the pharmacy for convenience and, in case of providers, increase profits. In the Pilot Extension study, pharmacy providers spent ~ 45 min per client delivering PrEP and PEP services. In Kenya, many pharmacies are operated by a single provider per shift, which makes it challenging to deliver time-consuming PrEP/PEP services during peak business hours when the priority is to serve many clients and maximize profits. In the original Pharm PrEP Pilot study that preceded the extension, pharmacy providers expressed frustration with the disruption that PrEP delivery caused to their regular workflow [9]. Additionally, studies among community pharmacists in Rwanda and Ghana found that while these professionals perceive public health activities positively, their engagement in such activities is often limited by time constraints and competing responsibilities [30, 31]. To facilitate the scale-up of pharmacy-delivered PrEP and PEP services, additional research is needed to identify implementation strategies—such as unassisted HIVST or telehealth visits with remote nurses—that reduce burden on pharmacy providers and clients through task-sharing, streamlining, and/or simplifying delivery components [12].

Our study had several limitations. First, we used data from two different pilot studies and the pharmacy providers reporting willingness to charge did not have any first-hand experience delivering PrEP and PEP; unfortunately, provider surveys were outside the scope of the Pilot Extension study that delivered pharmacy PrEP/PEP. Second, to estimate the time it took to deliver core components of the intervention, we relied on provider self-report rather than alternative methods that may have a smaller margin of error and are less subject to recall bias, such as direct observation; nevertheless, when we varied the amount of provider time in sensitivity analyses, our cost estimates remained robust. Third, our costing data was collected within the confines of a research study and may not be reflective of programmatic implementation. Fourth, the pharmacies that participated in both pilot studies were selected purposively and are not necessarily representative of all pharmacies in that county or Kenya broadly, thus limiting the generalizability of our findings. Finally, because clients in both pilot studies received PrEP/PEP services for free, their responses about willingness to pay for these services were anchored to a hypothetical scenario and might not reflect what actual willingness to pay would look like in a real-world scenario. Additionally, more rigorous approaches to willingness to pay could provide deeper insights. Future research should model the costs and savings of scaling pharmacy-based PrEP and PEP delivery, assessing sustainability, and cost-effectiveness for national HIV prevention strategies.

## Conclusion

Pharmacy-delivered PrEP and PEP services present a low-cost solution that could potentially expand PrEP and PEP coverage in Kenya, especially when subsidized with commodities from government stock. To effectively scale HIV prevention services in Kenya and similar contexts, the private sector, especially private pharmacies, must play a larger role in increasing access, particularly for individuals who can afford and are willing to pay for these services. This study provides key insights on the cost of PrEP and PEP delivery in a novel implementation setting, which can help inform the scale-up of pharmacy-delivered PrEP and PEP in Kenya and similar settings.

## Supplementary Information

Below is the link to the electronic supplementary material.

Supplementary Material 1

Supplementary Material 2

## Data Availability

All data generated or analyzed during this study are available upon request.
