# Supplementary material for: Understanding the cost of pharmacy-delivered HIV pre- and post-exposure prophylaxis service delivery in Kenya: findings from pilot studies
Source: BMC Health Serv Res. 2025 Nov 26;25:1536. doi: 10.1186/s12913-025-13681-x (PMC12659100; doi:10.1186/s12913-025-13681-x)
Supplement: Supplementary file 1 — Supplementary Material 1 [file 12913_2025_13681_MOESM1_ESM.docx]

**Additional file 1**

1. **Description of cost categories**

*Capital cost:* These were resources utilized for pharmacy services that remained the same even after the introduction of PrEP and PEP services. These included computers, printers, tables, chairs, pill counter plates, cabinets, stools, annual pharmacy licenses, annual pharmacist licenses, annual administrator licenses, and rent and safety boxes. We obtained their 2022 value and divided it by 2 to get the 6-month value representing the time the services were implemented. These costs were then directly allocated to PrEP & PEP services, based on the estimated percentage of pharmacy time spent on delivering these services. The estimated percentage of pharmacy time spent on delivering the new services was calculated as follows:

$$\% pharmacy time spent delivering services=\frac{Total time spent on the new services over 6 months}{Total pharmacy operation time over 6 months} x 100$$

NB: We assumed that pharmacies operate 12 hours a day from Monday to Saturday.

*Training:* Training of pharmacy providers (pharmacists and pharmaceutical technologists) and research assistants (RAs) took place over 2 days. We estimated the cost of the meeting hall for the 2 days, the training package and transport reimbursement for 12 pharmacy providers and 12 research assistants (RAs). We also estimated the daily pay of the training facilitator for the 2 days of training. We excluded the cost of training RAs since that will not apply in the real world scenario.

*Demand creation:* This included the expenses associated with designing, printing and distributing the Frequently Asked Questions (FAQ) flyers, which featured three different FAQs with images of a boy, a doctor, and a girl. Additionally, the cost covered the printing of study-specific posters and flyers. These expenses were allocated directly to PrEP & PEP services, based on the percentage of PrEP or PEP client visits within the study.

*Overhead cost:* These comprised resources utilized for pharmacy services that underwent changes following the introduction of PrEP and PEP services. These included registers, disposable cups, maintenance and repairs, utilities, medication vials/caps, gloves, pharmacy bags, receipt papers, masks, hand sanitizers, medical envelopes, and soap. These costs were directly allocated to PrEP and PEP services, determined by the estimated percentage of pharmacy time spent on delivering these services.

*Personnel:* Pharmacy personnel included pharmacists, pharmaceutical technologist(s), pharmacy assistants or nurses involved in PrEP and PEP services in the pharmacy. We derived the cost per minute of the personnel from their annual salary after subtracting time spent on lunch break, sick leave, and vacation. As shown in Table 1, we interviewed the pharmacy personnel to obtain the average time spent on the following aspects of PrEP and PEP services: screening, counselling, testing, dispensing, follow-up counselling, and record keeping. We excluded the time spent on research activities (e.g., consenting and DBS sample collection). We also estimated the cost of a remote clinician serving the 12 pharmacies involved in the PrEP delivery. Since the effort of the remote clinician was not documented during the project implementation, we assumed that a physician working at 5% full-time equivalent will be sufficient to attend to any query raised by the pharmacy providers from the participating pharmacies during the PrEP implementation.

Administrative staff included the pharmacy manager, cleaner, health record officer and cashier. Not all the pharmacies had these staff. The daily costs of these administrative staff were derived from their annual salary and then directly allocated to PrEP and PEP testing services, based on the estimated percentage of pharmacy time spent on delivering these services.

*Testing and drugs:* The cost of HIV Rapid Diagnostic Tests (RDT) was derived from Jhipiego procurement data, incorporating Value Added Tax (16% of original cost) and wastage (5% of resulting cost). The cost of PrEP and PEP medications was determined based on KEMSA competitive pricing, which may vary slightly with different procurement instances. The cost included considerations for warehousing and the distribution of the medication to medical stores.

*Other supplies:* These included other items for administrative purposes in the pharmacy and included a water dispenser, liquid soap, staplers, paper punch, stamps, box files, hand wash, staple pins, timer, and batteries. Since these items are shared by other services in the pharmacy, they were directly allocated to PrEP and PEP services, based on the estimated percentage of pharmacy time spent on delivering these services.

1. **Number of clients visits**

| PrEP initiation service | | *Month 1* | 141 |  |
| --- | --- | --- | --- | --- |
|  |  | Month 2 | 195 |  |
|  |  | Month 3 | 94 |  |
|  |  | Month 4 | 168 |  |
|  |  | Month 5 | 93 |  |
|  |  | Month 6 | 0 |  |
|  |  | **Total** | **691** |  |
| PrEP continuation service | | *Month 1* | 0 |  |
|  |  | Month 2 | 104 |  |
|  |  | Month 3 | 110 |  |
|  |  | Month 4 | 134 |  |
|  |  | Month 5 | 268 |  |
|  |  | Month 6 | 60 |  |
|  |  | **Total** | **676** |  |
| PEP service | | *Month 1* | 25 |  |
|  |  | Month 2 | 49 |  |
|  |  | Month 3 | 35 |  |
|  |  | Month 4 | 41 |  |
|  |  | Month 5 | 31 |  |
|  |  | Month 6 | 116 |  |
|  |  | **Total** | **197** | |
|  |  | **Grand total** | **1564** | |

1. **The unit value of resources**

| **Key parameters** | **Data** | **Data source** |
| --- | --- | --- |
| Average exchange rate in 2022 | 0.0086 | <https://www.exchangerates.org.uk/KES-USD-spot-exchange-rates-history-2022.html> |
| Consumer Price Index (Price inflator) | Various | World bank |
| Discount rate | 3% | Haacker, 2020^a^ |
| Useful life years for equipment and training | 5 years | Booth et al., 2018^b^ |
| Pharmacy time used for PrEP initiation | 3.0% | Estimated |
| Pharmacy time used for PrEP continuation | 1.5% | Estimated |
| Pharmacy time used for PEP visit | 0.6% | Estimated |
| HIV blood-based test kit wastage | 5% | Assumption |
| Oral PrEP (1 bottle, 30 tablets), USD^1^ | 5.9 | KEMSA competitive pricing |
| Oral PEP (1 bottle, 30 tablets), USD^1^ | 3.5 | KEMSA competitive pricing |
| HIV blood-based rapid diagnostic test kit^2^, USD | 3.6 | Jhipiego procurement data |
| Remote clinical salary per month, KES^3^ | 180,000 | Glassdoor.com |
| Pharmacy operation time, hours | 12 | Assumption |
| Workdays per week, days | 6 | Ortblad, 2023^c^ |
| **Training** | **Cost (KES)** | **Data source** |
| Meeting hall for about 100 people | 4000 | Advertised hall at jiji.com |
| Training package/trainee | 2200 | Data provided by Jhpiego |
| Transport reimbursement/trainee | 1000 | Data provided by Jhpiego |
| Daily pay for training facilitator | 1516.7 | Monthly pay for laboratory technician obtained from Glassdoor. |

^1^Includes warehousing and distribution

^2^Incudes 5% wastage

^3^Effort on project estimated at 5% full-time equivalent

1. **Costing Questionnaire**

# **Pharmacy PrEP Costing Form**

| This costing form is to be completed by Pharmacy PrEP staff/pharmacy staff/research assistants in respective pharmacies regarding overarching costs of the cRCT. Staff/research assistants should do their best to ensure responses to all questions and may enter the data on behalf of the informant. Thank you. |
| --- |

| **0. Demographics** | | | |
| --- | --- | --- | --- |
| Your name (to be deidentified, tracking purpose only): | | | *Name: ______________________________* |
| Role: | | | *Research Assistant (RA)*  *Pharmacist*  *Pharmacy technician*  *Pharmacy owner*  *Laboratory staff*  *Other_____________________________* |
| Site: | | | *Nairobi*  *Kiambu*  *Kisumu*  *Homa Bay* |
| Pharmacy name: | | | *____________________________________* |
| Date filled out | | | *\|__\|__\|__\|__\|__\| __\| number (DD/MM/YY)* |
| **A. Start-up costs** | | |  |
| 1 | **How much does equipment in the pharmacy cost for the following items:** |  |  |
|  | - Registers | *________ KSh (each) x _______ quantity,*  *________ Year purchased* |  |
|  | - Computers | *________ KSh (each) x _______ quantity,*  *________ Year purchased* |  |
|  | - Printers | *________ KSh (each) x _______ quantity,*  *________ Year purchased* |  |
|  | Furniture: |  |  |
|  | - Tables | *________ KSh (each), x _______ quantity,*  *________ Year purchased* |  |
|  | - Chairs | *________ KSh (each), x _______ quantity,*  *________ Year purchased* |  |
|  | - Other furniture: ________ | *________ KSh (each), x _______ quantity,*  *________ Year purchased* |  |
|  | - Pill counter plates | *________ KSh (each) x _______ quantity,*  *________ Year purchased* |  |
|  | - Other | *________ KSh per _______*  *________ Year purchased* |  |
| 2 | How much does pharmacy facility licensure cost annually? | *________ KSh* |  |
| 3 | How much does pharmacist licensure cost annually? | *________ KSh* |  |
| 4 | How much does it cost to pay administrators/ supervisors of the pharmacy per month? | *________ KSh* |  |
| **B. Overhead costs** | | |  |
| 5 | About how much does the pharmacy spend on maintenance and repairs? Ex. sewage, electricity, painting, infrastructure) | *________ KSh (per _______________)(ex. month, year) for _______________ (service)*  *________ KSh (per _______________)(ex. month, year) for _______________ (service)*  *________ KSh (per _______________)(ex. month, year) for _______________ (service)*  *________ KSh (per _______________)(ex. month, year) for _______________ (service)* |  |
| 6 | How much does it cost to rent the facility space per month (including taxes)? If the facility space is not rented, what is the estimated rental cost? | *________ KSh* |  |
| 7 | About how much does the pharmacy spend on utilities per month? (e.g., water, electricity)? | *________ KSh* |  |
| 8 | Please review and add to any materials used for the study that is not listed below: |  |  |
|  | - Medication vials/caps | *________ KSh (per box, count = _____)*  *________ (approximate # used per month)* |  |
|  | - Gloves | *________ KSh (per box, count = _____)*  *________ (approximate # used per month)* |  |
|  | - Pharmacy bags | *________ KSh (per box, count = _____)*  *________ (approximate # used per month)* |  |
|  | - Receipt paper | *________ KSh (per box, count = _____)*  *________ (approximate # used per month)* |  |
|  | - *Other: ____________________* | *________ KSh (per _______________)*  *________ (approximate # used per month)* |  |
|  | - *Other: ____________________* | *________ KSh (per _______________)*  *________ (approximate # used per month)* |  |
| 9 | Please review and add to any materials used specifically due to COVID-19/related PPE specific to COVID-19 that are given by the pharmacy for the public to use: |  |  |
|  | - Masks | *________ KSh (per _______________)*  *________ (approximate # used per month)* |  |
|  | - Hand sanitizer | *________ KSh (per _______________)*  *________ (approximate # used per month)* |  |
|  | - *Other: ____________________* | *________ KSh (per _______________)*  *________ (approximate # used per month)* |  |
|  |  |  |  |
| **C. Personnel costs** | | | |
| 10 | What unpaid individuals work in the pharmacy? | **[Job A]:** *Other: ____________________*  **[Job B]:** *Other: ____________________*  **[Job C]:** *Other: ____________________* |  |
| 11 | What paid individuals work in the pharmacy? | **[Job 1]:** *Pharmaceutical technologist*  **[Job 2]:** *Other: ____________________*  **[Job 3]:** *Other: ____________________* |  |
| 12 | Which workers are involved in the delivery of PrEP at pharmacies? | **[Job 1]:** *Pharmaceutical technologist*  **[Job 2]:** *Other: ____________________*  **[Job 3]:** *Other: ____________________*  **[Job A]:** *Other: ____________________*  **[Job B]:** *Other: ____________________*  **[Job C]:** *Other: ____________________* |  |
| 13 | How much does it cost (per year) to hire each worker?  ***NOTE: only include those involved in pharmacy PrEP delivery here. Details such as advertisement costs should be included in the discussion.*** | **[Job 1]:** *________ KSh/year*  **[Job 2]:** *________ KSh/year*  **[Job 3]:** *________ KSh/year* |  |
| 14 | How many hours does each worker work per day? | **[Job A]:** *________ hours*  **[Job B]:** *________ hours*  **[Job C]:** *________ hours*  **[Job 1]:** *________ hours*  **[Job 2]:** *________ hours*  **[Job 3]:** *________ hours* |  |
| 15 | **How long are breaks (for lunch, etc please specify)?** | **[Job A]:** *________ minutes/day*  **[Job B]:** *________ minutes/day*  **[Job C]:** *________ minutes/day*  **[Job 1]:** *________ minutes/day*  **[Job 2]:** *________ minutes/day*  **[Job 3]:** *________ minutes/day* |  |
| 16 | **How much total vacation does each worker have? Please note how it is accrued/rate.** | **[Job 1]:** *________ hours per ___________*  **[Job 2]:** *________ hours per ___________*  **[Job 3]:** *________ hours per ___________* |  |
| 17 | **How much sick leave does each worker have? Please note how it is accrued/rate.** | **[Job 1]:** *________ hours per ___________*  **[Job 2]:** *________ hours per ___________*  **[Job 3]:** *________ hours per ___________* |  |
| 18 | **How many paid holidays do you work in a given year?** | **[Job 1]:** *________ days*  **[Job 2]:** *________ days*  **[Job 3]:** *________ days* |  |
| 19 | **Are there any other benefits that workers receive? (Ex. insurance, subsidized meals, pension etc discussed in person)** | **[Job ___]:** *________________ benefits (please describe and include quantity)*  **[Job ___]:** *________________ benefits (please describe and include quantity)*  **[Job ___]:** *________________ benefits (please describe and include quantity)* |  |
| 20 | About how long does it take to enroll a participant in the cRCT? | *________ minutes* |  |
| 21 | About how long does the PrEP initiation visit last (including screening, counseling, testing, dispensing and recordkeeping)? | *________ minutes* |  |
| 22 | About how long does the PrEP continuation visit last (including screening, counseling, testing, dispensing, and recordkeeping)? | *________ minutes* |  |
| 23 | About how long does the PEP visit last (including screening, counseling, testing, dispensing, follow-up and recordkeeping)? | *________ minutes* |  |
| 24a | About what percent of each staff member’s total time in the pharmacy is spent on PrEP-related activities? | **[Job A]:** *________ %*  **[Job B]:** *________ %*  **[Job C]:** *________ %*  **[Job 1]:** *________ %*  **[Job 2]:** *________ %*  **[Job 3]:** *________ %* |  |
| 24b | Based on your best estimate, what percent of pharmacy time is used to deliver PrEP?  (E.g., if a pharmacy operates 14hrs/day & 3hrs/day is dedicated to PrEP related activities, then 14.2% of pharmacy time is dedicated to PrEP delivery) | *________ %* |  |
| **D. PrEP appointment/missed visit reminders** | | | |
| 25 | Does this pharmacy call PrEP clients to remind them about their appointments? | *Yes*  *No* |  |
| 26 | Who makes the reminder calls? | *Pharmaceutical technologist*  *Research assistant*  *Other: ________* |  |
| 27 | How long are the calls typically? | *________ minutes* |  |
| 28 | Does this pharmacy call PrEP clients if they miss their appointments? | *Yes*  *No* |  |
| 29 | Who makes the missed visit calls? | *Pharmaceutical technologist*  *Research assistant*  *Other: ________* |  |
| 30 | How long are the calls typically? | *________ minutes* |  |
| **F. PrEP delivery supplies** | | | |
| ***PrEP medication*** | | |  |
| 31 | What is the wholesale acquisition cost for PrEP medication through your supplier? | *________ KSh (per _____# bottle/s)* |  |
| 32 | How much PrEP medication expired before it was able to be dispensed at the pharmacy (outdated + wasted)? | *________ number bottles per _______ (period of time, e:x year)* |  |
| ***HIV self-tests*** | | |  |
| 33 | What is the wholesale acquisition cost of an HIV self-test kit through your supplier? | **[Oral-fluid kit]:** *________ KSh (per kit)*  **[Blood-based kit]:** *________ KSh (per kit)*  *N/A* |  |
| 34 | How much do you charge clients for an HIV self-test kit at your pharmacy? | **[Oral-fluid kit]:** *________ KSh (per kit)*  **[Blood-based kit]:** *________ KSh (per kit)*  *N/A* |  |
| 35 | How long does it take to complete the provider-assisted HIV self-testing process? | __________ minutes |  |
| 36 | How long does it take to complete the provider-unassisted HIV self-testing process? | __________ minutes  *N/A* |  |
| ***HIV rapid tests*** | | |  |
| 37 | What is the wholesale acquisition cost of an HIV rapid kit through your supplier? | *________ KSh (per test)*  *N/A* |  |
| 38 | How much do you charge clients for an HIV rapid test at your pharmacy? | *________ KSh (per test)*  *N/A* |  |
| 39 | How long does it take to complete the rapid HIV testing process? | __________ minutes  *N/A* |  |
| **G. STI Screening Visit** | | |  |
| 40 | What is the wholesale acquisition cost for Urine cups and how many were used? | *________ KSh/per cup/ _____ # of cups* |  |
| 41 | What is the wholesale acquisition cost for the cooler box and how many were used? | *________ KSh/per cooler box/ _____ # of cooler box* |  |
| 42 | What is the wholesale acquisition cost for the tissue papers and how many were used? | *________ KSh/per tissue paper/ _____ # of tissue paper* |  |
| 43 | What is the cost of transporting the cooler box to the laboratory and how many did you transport? | *________ KSh/per transport of cooler box/ _____ # of times transport occurred* |  |
| 44 | What is the cost of STI testing and how many STI test did you perform? | *________ KSh/per STI test/ _____ # of STI test* |  |
| **G. Miscellaneous** | | |  |
| 45 | Any other costs we haven’t mentioned that you believe would pertain to pharmacy-initiated PrEP? Please describe in detail below: |  |  |

1. **Time and Motion Study Questionnaire**

# **Pharmacy PrEP Time and Motion Study**

| This form is to be completed by research assistants/pharmacy staff in respective pharmacies **after observing** the time spent on PrEP-related activities and the equipment used to perform the activities. Kindly fill in all sections accurately. Thank you. |
| --- |

| **0. Demographics** | | | | |
| --- | --- | --- | --- | --- |
| Your name (to be deidentified, tracking purpose only): | | | *Name: ______________________________* | |
| Role: | | | *Research Assistant (RA)*  *Pharmacist*  *Pharmacy technician*  *Pharmacy owner*  *Laboratory staff*  *Other_____________________________* | |
| Site: | | | *Nairobi*  *Kiambu*  *Kisumu*  *Homa Bay* | |
| Pharmacy name: | | | *________________________________* | |
| Visit types completed at this site (check all that apply): | | | *PrEP initiation visit*  *PrEP continuation (follow-up) visit*  *PEP visit* | |
| Observation date | | | *\|__\|__\|__\|__\|__\| __\| number (DD/MM/YY)* | |
| **Instructions for all activities**   1. Fill out sections A-D and list all the activities below as needed (for examples, please see the example sheet). Document in detail. 2. For each subcategory, record the start time and the end time (e.g., 1:30 PM)   **Please note**: If the task you are observing is interrupted, please note the time you paused and restarted. We are specifically interested in an active time of tasks (i.e., not pauses, breaks, or interruptions such as waiting for an HIVST test). Thank you! | | | | |
|  |  |  |  |  |
| 1. **PrEP Initiation Visit**   Please fill out all activities from when a client enters for a PrEP initiation visit. Please include the activity description in detail, equipment/tool and quantity used for the activity, and record the start time and the end time (e.g., 1:30 PM).  Activity subcategories: (activities listed below will generally fall into one of these types of categories)   1. Screening: Includes assessing client eligibility, RAST assessment, medical safety questionnaire, etc. 2. Counseling: Includes pre- and post-HIVST counseling, PrEP counseling, any remote clinician contact, etc. 3. Testing: Includes conducting actual HIVST and test wait time, etc. 4. Dispensing: Includes preparation of PrEP, preparation and/or completion of PrEP/PEP register, etc. 5. Recordkeeping: Includes updating records for MOH commodities/supply chain management, etc. | | | |  |
| 1 | Activity description: | *Equipment used (units)* | *Time stop: _________*  *Time start:_________* |  |
| 2 | Activity description: | *Equipment used (units)* | *Time stop: _________*  *Time start:_________* |  |
| 3 | Activity description: | *Equipment used (units)* | *Time stop: _________*  *Time start:_________* |  |
| 4 | Activity description: | *Equipment used (units)* | *Time stop: _________*  *Time start:_________* |  |
| 5 | Activity description: | *Equipment used (units)* | *Time stop: _________*  *Time start:_________* |  |
| 6 | Activity description: | *Equipment used (units)* | *Time stop: _________*  *Time start:_________* |  |
| 7 | Activity description: | *Equipment used (units)* | *Time stop: _________*  *Time start:_________* |  |
| 8 | Activity description: | *Equipment used (units)* | *Time stop: _________*  *Time start:_________* |  |
| 9 | Activity description: | *Equipment used (units)* | *Time stop: _________*  *Time start:_________* |  |
| 10 | Activity description: | *Equipment used (units)* | *Time stop: _________*  *Time start:_________* |  |
| 11 | Activity description: | *Equipment used (units)* | *Time stop: _________*  *Time start:_________* |  |
| 12 | Activity description: | *Equipment used (units)* | *Time stop: _________*  *Time start:_________* |  |
| 13 | Activity description: | *Equipment used (units)* | *Time stop: _________*  *Time start:_________* |  |
| 14 | Activity description: | *Equipment used (units)* | *Time stop: _________*  *Time start:_________* |  |
| 15 | Activity description: | *Equipment used (units)* | *Time stop: _________*  *Time start:_________* |  |
| **If more activities, please print the extra activity sheet and continue.* | | | |  |
|  |  |  |  |  |
| 1. **PrEP Continuation Visit**   Please fill out all activities from when a client enters for a PrEP continuation visit. Please include the activity description in detail, equipment/tool and quantity used for the activity, and record the start time and the end time (e.g., 1:30 PM).  Activity subcategories: (activities listed below will generally fall into one of these types of categories)   1. Screening: Includes risk assessment, etc. 2. Counseling: Includes pre- and post-HIVST counseling, PrEP counseling, any remote clinician contact, etc. 3. Testing: Includes conducting actual HIVST and test wait time, etc. 4. Dispensing: Includes preparation of PrEP, preparation and/or completion of the register, etc. 5. Recordkeeping: Includes updating records for MOH commodities/supply chain management, etc. | | | |  |
| 1 | Activity + equipment needed below: | *Equipment used (units)* | *Time stop: _________*  *Time start:_________* |  |
| 2 | Activity + equipment needed below: | *Equipment used (units)* | *Time stop: _________*  *Time start:_________* |  |
| 3 | Activity + equipment needed below: | *Equipment used (units)* | *Time stop: _________*  *Time start:_________* |  |
| 4 | Activity + equipment needed below: | *Equipment used (units)* | *Time stop: _________*  *Time start:_________* |  |
| 5 | Activity + equipment needed below: | *Equipment used (units)* | *Time stop: _________*  *Time start:_________* |  |
| 6 | Activity + equipment needed below: | *Equipment used (units)* | *Time stop: _________*  *Time start:_________* |  |
| 7 | Activity + equipment needed below: | *Equipment used (units)* | *Time stop: _________*  *Time start:_________* |  |
| 8 | Activity + equipment needed below: | *Equipment used (units)* | *Time stop: _________*  *Time start:_________* |  |
| 9 | Activity + equipment needed below: | *Equipment used (units)* | *Time stop: _________*  *Time start:_________* |  |
| 10 | Activity + equipment needed below: | *Equipment used (units)* | *Time stop: _________*  *Time start:_________* |  |
| 11 | Activity + equipment needed below: | *Equipment used (units)* | *Time stop: _________*  *Time start:_________* |  |
| 12 | Activity + equipment needed below: | *Equipment used (units)* | *Time stop: _________*  *Time start:_________* |  |
| 13 | Activity + equipment needed below: | *Equipment used (units)* | *Time stop: _________*  *Time start:_________* |  |
| 14 | Activity + equipment needed below: | *Equipment used (units)* | *Time stop: _________*  *Time start:_________* |  |
| 15 | Activity + equipment needed below: | *Equipment used (units)* | *Time stop: _________*  *Time start:_________* |  |
| **If more activities, please print the extra activity sheet and continue.* | | | |  |
|  |  |  |  |  |
| 1. **PEP Visit**   Please fill out all activities from when a client enters for a PEP visit. Please include the activity description in detail, equipment/tools and quantity used for the activity, and record the start time and the end time (e.g., 1:30 PM).  Activity subcategories: (activities listed below will generally fall into one of these types of categories)   1. Screening: Includes PEP assessment, RAST assessment, medical safety questionnaire, etc. 2. Counseling: Includes pre- and post-HIVST counseling, PEP counseling, any remote clinician contact, etc. 3. Testing: Includes conducting actual HIVST and test wait time, etc. 4. Dispensing: Includes preparation of PEP, preparation and/or completion of the register, etc. 5. Follow-up: Includes follow-up counseling, including processes if a client transitions to PrEP, etc. 6. Recordkeeping: Includes updating records for MOH commodities/supply chain management, etc. | | | |  |
| 1 | Activity + equipment needed below: | *Equipment used (units)* | *Time stop: _________*  *Time start:_________* |  |
| 2 | Activity + equipment needed below: | *Equipment used (units)* | *Time stop: _________*  *Time start:_________* |  |
| 3 | Activity + equipment needed below: | *Equipment used (units)* | *Time stop: _________*  *Time start:_________* |  |
| 4 | Activity + equipment needed below: | *Equipment used (units)* | *Time stop: _________*  *Time start:_________* |  |
| 5 | Activity + equipment needed below: | *Equipment used (units)* | *Time stop: _________*  *Time start:_________* |  |
| 6 | Activity + equipment needed below: | *Equipment used (units)* | *Time stop: _________*  *Time start:_________* |  |
| 7 | Activity + equipment needed below: | *Equipment used (units)* | *Time stop: _________*  *Time start:_________* |  |
| 8 | Activity + equipment needed below: | *Equipment used (units)* | *Time stop: _________*  *Time start:_________* |  |
| 9 | Activity + equipment needed below: | *Equipment used (units)* | *Time stop: _________*  *Time start:_________* |  |
| 10 | Activity + equipment needed below: | *Equipment used (units)* | *Time stop: _________*  *Time start:_________* |  |
| 11 | Activity + equipment needed below: | *Equipment used (units)* | *Time stop: _________*  *Time start:_________* |  |
| 12 | Activity + equipment needed below: | *Equipment used (units)* | *Time stop: _________*  *Time start:_________* |  |
| 13 | Activity + equipment needed below: | *Equipment used (units)* | *Time stop: _________*  *Time start:_________* |  |
| 14 | Activity + equipment needed below: | *Equipment used (units)* | *Time stop: _________*  *Time start:_________* |  |
| 15 | Activity + equipment needed below: | *Equipment used (units)* | *Time stop: _________*  *Time start:_________* |  |
| **If more activities, please print the extra activity sheet and continue.* | | | |  |

1. **Willingness to Pay or Provide Questions**

| ***PrEP*** |  |
| --- | --- |
| ***Willingness to pay*** |  |
| The Kenya Ministry of Health (MOH) and the organizations funding this research study are covering the cost of PrEP services so that participants like you do not have to pay anything for PrEP. However, in the future, to make delivering PrEP profitable for pharmacies, pharmacies might be allowed to charge clients for PrEP. In the future, how much might you be willing to pay to receive HIV testing, counseling, and a 3-month supply of PrEP at a pharmacy like this one? | *Average: _____ KSh* |
| ***Willingness to provide*** |  |
| If your pharmacy received PrEP drugs and HIVST kits for free from the Ministry of Health, what is the maximum amount you would charge clients each time they came to the pharmacy for PrEP?  *(Include in your estimate any cost you would charge for assessing clients’ HIV risk, helping clients with the HIVST if needed, counseling, and drug storage and dispensing.)* | *Maximum: _____ KSh* |
| What is the minimum amount you would charge clients each time they came to the pharmacy for PrEP? | *Minimum: _____ KSh* |
| *Imagine that the Ministry of Health is willing to compensate you for delivering PrEP at your pharmacy. In this scenario, you will confirm that clients are HIV-negative and thus eligible for PrEP using the results of HIV self-tests that clients conduct on their own in a back room of your pharmacy. At times, you may have to help clients conduct these self-tests. Imagine that the Ministry of Health is providing you with the PrEP drugs and HIVST kits for free.*   - In this scenario, would you prefer the Ministry of Health to pay you a **fixed amount of money each month** for providing PrEP regardless of how many PrEP clients you serve, or compensate using a **fee-for-service model** where the amount of money paid per month is based on the number of PrEP clients you serve? | *Monthly compensation*  *Fee-for-service*  *Other compensation model: ______________________* |
| ***PEP*** |  |
| ***Willingness to provide*** |  |
| If your pharmacy received the PEP drugs and HIVST kits for free from the Ministry of Health, what is the maximum amount you would charge clients for PEP delivery?  *(Include in your estimate any cost you would charge for assessing clients’ HIV risk, helping clients with the HIVST if needed, counseling, and drug storage and dispensing.)* | *Maximum: _____ KSh* |
| What is the minimum amount you would charge clients for PEP delivery? | *Minimum: _____ KSh* |
| *Imagine that the Ministry of Health is willing to compensate you for delivering PEP at your pharmacy and will provide the PEP drugs and HIVST for free. For PEP delivery you will counsel clients, confirm they are HIV-negative (using HIV self-testing) and PEP eligible (using a prescribing checklist).*  In this scenario, would you prefer the Ministry of Health to pay you a **fixed amount of money each month** for providing PEP regardless of how many PrEP clients you serve, or compensate using a **fee-for-service model** where the amount of money paid per month is based on the number of PEP clients you serve? | *Monthly compensation*  *Fee-for-service*  *Other compensation model: ______________________* |
| ***Injectable PrEP*** |  |
| If your pharmacy received all of the supplies needed to deliver injectable PrEP, as well as a trained nurse, for free from the Ministry of Health, what is the maximum amount you would charge clients for **each visit**?  *(Include in your estimate any costs associated with , properly storing the injection, counseling clients, administering the injection, and reporting number of injections administered to the Ministry of Health.)* | *Maximum: _____ KSh* |
| What is the minimum amount you would charge clients for **each visit**? | *Minimum: _____ KSh* |
| *Imagine that the Ministry of Health is willing to compensate you for providing injectable PrEP at your pharmacy and will provide your pharmacy with the necessary supplies for delivering injectable PrEP.*   - In this scenario, would you prefer the Ministry of Health to pay you a **fixed amount of money each month** for providing injectable PrEP regardless of how many clients you attend to, or compensate using a **fee-for-service model** where the amount of money paid per month is based on the number of clients you attend to? | *Monthly compensation*  *Fee-for-service*  *Other compensation model: ______________________* |

1. **Subsidized delivery**
2. **Non-subsidized delivery**

**Figure 1. Impact of various service offering scenarios on the economic cost per person-month on PrEP and PEP**

1. **Subsidized delivery**
2. **Nonsubsidized delivery**

**Figure 2. Impact of various service offering scenarios on the financial cost per person-month on PrEP and PEP**

**Figure 3. Impact of standardized labor practices on the financial cost per person-month on PrEP and PEP**

**References**

a. Haacker, M., Hallett, T. B. & Atun, R. On discount rates for economic evaluations in global health. *Health Policy and Planning* czz127 (2019) doi:10.1093/heapol/czz127.

b. Booth, N. *et al.* Costs of screening for prostate cancer: Evidence from the Finnish Randomised Study of Screening for Prostate Cancer after 20-year follow-up using register data. *European Journal of Cancer* **93**, 108–118 (2018).

c. Ortblad KF, Kwach B, Zhang S, Asewe M, Ongwen PA, Malen RC, et al. Measuring the performance of HIV self‐testing at private pharmacies in Kenya: a cross‐sectional study. J Int AIDS Soc. 2023 Oct;26(10):e26177.

**Additional file 2**

Costing template
